# 

**Published:** 1893-06-03

**Authors:** 


**OUR NEW OFFICES.**
428, Strand, W.C., will henceforth be the Office of this Journal.

**Notices of Births, Deaths, and Marriages,** are inserted in this column at a charge of 2s. 6d. for an announcement not exceeding four lines.

### NOTICE TO CORRESPONDENTS.

All MS., Letters, Books for Review, and other matters intended for the Editor should be addressed The Editor, The Lodge, Porchester Square, London, W.

The Editor cannot undertake to return rejected M.S., even when accompanied by stamped directed envelope.

**Notice to Advertisers and Subscribers.**

All Advertisements, Orders for Copies of The Hospital, and Business Communications should be addressed to The Publisher, *not to the Editor*, The Hospital, 428, Strand, W.C.
The Cost of Subscription, post free, is as follows:—Per week, 2½d.; per quarter, 2s. 6d.; per half-year, 5s.; per annum, 8s. 6d. Foreign:—Per annum, 10s. 8d.; payable, in all cases, in advance.

**"Burdett's Hospital Annual," 1893.**

Fourth year of publication. 700 pp. Boards, gilt lettering. A most complete guide to British, Colonial, Indian, and American Hospitals, Dispensaries, Nursing and Convalescent Institutions, and Asylums. Price 5s. Published by The Scientific Press (Limited), 428, Strand, W.C.

**NOTICE.—The Index for Vol. XIII. (ending March 1893) is on sale at the Office of this Journal, price 2½d., post free.**

### A YEAR'S WORK IN THE HOSPITALS AND MEDICAL CHARITIES OF LONDON.

The following figures relate to the year 1892, and have been prepared in the office of the Metropolitan Hospital Sunday Fund :—

### NEWINGTON AND SOUTH DISTRICT,

Comprising Battersea, Wandsworth, Tooting, Balham, Streatham, Brixton, Lambeth, Newington, Southwark, Bermondsey, Camberwell, Greenwich, Deptford, Lewisham, Blackheath, Woolwich, &c.

| No. of Beds. | No. of Beds Daily Occupied. | HOSPITALS. | In-patients. | Out-patients. | Total Expenditure. | Income. | | | Total Income. | Legacies not included in previous column. |
|---|---|---|---|---|---|---|---|---|---|---|
| | | | | | | Charitable. | Proprietary. | Patients' Payments. | | |
| | | | | | £ | £ | £ | £ | £ | £ |
| 600 | 433 | Guys' ... ... ... ... ... | 6,136 | 59,846 | 39.300 | 7,549 | 27,493 | 4,065 | 39,107 | ... |
| 23 | 20 | Miller... ... ... ... ... | 242 | 12,539 | 2 883 | 2,515 | 372 | ... | 2,887 | 300 |
| 253 | 219 | Seamen's ... ... ... ... | 2,690 | 13,745 | 15,212 | 7,471 | 3,881 | 151 | 11,503 | 709 |
| 66 | 51 | Evelina, for Children ... ... | 577 | 5,201 | 6,323 | 3,917 | 584 | 76 | 4,577 | 100 |
| 45 | 44 | Home for Sick Children ... ... | 218 | 1,395 | 1,595 | 1,282 | 55 | 306 | 1,643 | 342 |
| 24 | 20 | General Lying-In ... ... ... | 490 | 1,291 | 3,098 | 692 | 2,396 | ... | 3,088 | ... |
| 21 | 9 | Clapham Maternity ... ... | 206 | 370 | 930 | 432 | 264 | 372 | 1,068 | ... |
| 51 | 50 | Royal, for Children and Women... | 576 | 7,193 | 4,544 | 3,860 | 739 | 200 | 4,799 | 229 |
| Rebui | lding. | Royal Eye ... ... ... ... | ... | 9,076 | 1,017 | 916 | 81 | 12 | 1,009 | ... |
| 10 | 3 | Hospital for Diseases of the Skin.. | 17 | 5,099 | 1,087 | 244 | 120 | 580 | 944 | 145 |
| 370 | 169 | Metropolitan Convalescent ... | 2,974 | ... | 6,469 | 5.076 | 620 | 60 | 5,756 | 1,530 |
| 1,463 | 1,018 | | 14,126 | 115,755 | 82,458 | 33,954 | 36,605 | 5,822 | 76,381 | 3,355 |
| | | DISPENSARIES. | | | | | | | | |
| ... | ... | Battersea, Provident ... ... | ... | 11,126 | 2,271 | 84 | 57 | 2,118 | 2,259 | ... |
| ... | ... | Brixton, &c. ... ... ... ... | ... | 12,729 | 851 | 519 | 58 | 115 | 692 | ... |
| ... | ... | Camberwell, Provident ... ... | ... | 11,438 | 1,949 | 475 | 104 | 1,412 | 1,991 | ... |
| ... | ... | Clapham ... ... ... ... | ... | 1,970 | 486 | 270 | ... | 134 | 404 | 100 |
| ... | ... | Forest Hill ... ... ... ... | ... | 2,270 | 582 | 211 | ... | 376 | 587 | ... |
| ... | ... | Gipsy Hill ... ... ... ... | ... | 1,130 | 184 | 50 | ... | 125 | 175 | ... |
| ... | ... | Royal South London ... ... | ... | 5,237 | 798 | 626 | 109 | ... | 735 | 100 |
| ... | ... | South Lambeth, &c. .. ... | ... | 2,231 | 542 | 364 | 17 | 181 | 562 | ... |
| ... | ... | South London Medical Aid ... | ... | 1,602 | 578 | 33 | 80 | 404 | 517 | ... |
| ... | ... | Wandsworth Common ... ... | ... | 740 | 197 | 63 | ... | 133 | 196 | ... |
| 1,463 | 1,018 | | 14,126 | 166,228 | 90,896 | 36,649 | 37,030 | 10,820 | 84,499 | 3,555 |

### CITY AND EAST CENTRAL DISTRICT,

Comprising the City, St. Luke's, Shoreditch, Finsbury, and Clerkenwell.

| No. of Beds. | No. of Beds Daily Occupied. | HOSPITALS. | In-patients. | Out-patients. | Total Expenditure. | Income. | | | Total Income. | Legacies not included in previous column. |
|---|---|---|---|---|---|---|---|---|---|---|
| | | | | | | Charitable. | Proprietary. | Patients' Payments. | | |
| | | | | | £ | £ | £ | £ | £ | £ |
| 78 | 66 | Metropolitan... ... ... ... | 845 | 16,325 | 9,149 | 3,103 | 465 | 774 | 4,342 | 2,927 |
| 160 | 125 | Royal Free ... ... ... ... | 1,757 | 23,381 | 10,550 | 3,860 | 1,259 | ... | 5,119 | 8,864 |
| 80 | 45 | Royal, for Diseases of the Chest ... | 542 | 6,231 | 6,636 | 4,372 | 119 | ... | 4,491 | 1,135 |
| 55 | 41 | North-Eastern, for Children ... | 556 | 14,233 | 5,183 | 223 | 2,862 | 750 | 3,835 | 107 |
| 36 | 24 | City of London Lying-In ... ... | 487 | 1,536 | 3,646 | 629 | 3,007 | 5 | 3,641 | 89 |
| 34 | 20 | St. Marks, for Fistula ... ... | 296 | 731 | 2,246 | 981 | 705 | ... | 1,686 | 5,940 |
| 100 | 83 | Royal London Ophthalmic ... | 1,967 | 26,495 | 7,538 | 2,801 | 880 | ... | 3,681 | 7,503 |
| 27 | 19 | City Orthopædic ... ... ... | 161 | 1,950 | 1,402 | 1,167 | 101 | ... | 1,268 | ... |
| 16 | 13 | Central London Throat and Ear ... | 288 | 6,250 | 2,256 | 707 | 23 | 1,302 | 2,032 | 470 |
| 586 | 436 | | 6,899 | 97,132 | 48,606 | 17,843 | 9,421 | 2,831 | 30,095 | 27,032 |
| | | DISPENSARIES. | | | | | | | | |
| ... | ... | City ... ... ... ... ... | ... | 4,610 | 1,303 | 1,255 | 190 | ... | 1,445 | ... |
| ... | ... | City of London and East London... | ... | 9,836 | 990 | 140 | 28 | 905 | 1,073 | ... |
| ... | ... | Farringdon General... ... ... | ... | 6,719 | 570 | 249 | 305 | ... | 554 | ... |
| ... | ... | Finsbury ... ... ... ... | ... | 12,647 | 873 | 665 | 129 | 265 | 1,059 | 50 |
| ... | ... | Metropolitan... ... ... ... | ... | 8,155 | 715 | 295 | 118 | 282 | 695 | ... |
| ... | ... | Royal General ... ... ... | ... | 3,674 | 973 | 778 | 335 | 102 | 1,215 | ... |
| 586 | 436 | | 6,899 | 142,773 | 54,030 | 21,225 | 10,526 | 4,385 | 36,136 | 27,082 |

### ST. MARYLEBONE AND WEST CENTRAL DISTRICT,

Comprising St. Marylebone, St. John's Wood, Bloomsbury, Holborn, &c.

| No. of Beds. | No. of Beds Daily Occupied. | HOSPITALS. | In-patients. | Out-patients. | Total Expenditure. | Income. Charitable. | Income. Proprietary. | Income. Patients' Payments. | Total Income. | Legacies not included in previous column. |
|---|---|---|---|---|---|---|---|---|---|---|
| | | | | | £ | £ | £ | £ | £ | £ |
| 60 | 44 | French ... ... ... ... ... | 652 | 5,096 | 3,470 | 3,470 | 53 | ... | 3,523 | 1,350 |
| 20 | 12 | Italian ... ... ... ... | 241 | 3 460 | 806 | 939 | 100 | ... | 1,039 | ... |
| 51 | 46 | SS. John and Elizabeth ... ... | 101 | ... | 1,751 | 1,546 | 769 | ... | 2,315 | ... |
| 320 | 254 | The Middlesex ... ... ... | 2,912 | 39,312 | 35 955 | 9,349 | 9,157 | ... | 18,506 | 31,035 |
| 81 | 79 | Alexandra, for Children ... ... | 147 | ... | 2 816 | 1,538 | 94 | 539 | 2,171 | 1,000 |
| 30 | 29 | Home for Incurable Children ... | 33 | ... | 1,213 | 629 | 7 | 477 | 1,113 | 760 |
| 182 | 120 | Hospital for Sick Children ... | 1,518 | 21,045 | 12,381 | 6,779 | 3,039 | ... | 9,818 | 5,096 |
| 16 | 8 | British Lying-In ... ... | 153 | 435 | 1,634 | 654 | 1,099 | 4 | 1,757 | 324 |
| 58 | 38 | Queen Charlotte's Lying-In ... | 972 | 1,133 | 3,923 | 2,618 | 365 | 8 | 2 991 | 410 |
| 42 | 36 | New Hospital for Women... ... | 500 | 9 484 | 3,449 | 2,493 | 173 | 1,063 | 3,729 | ... |
| 53 | 45 | Samaritan Free ... ... ... | 562 | 8 339 | 7,766 | 3,832 | 231 | ... | 4,063 | 2,569 |
| 170 | 157 | National for the Paralysed, &c. ... | 828 | 4,281 | 12,398 | 3,972 | 1,554 | 2,278 | 7,804 | 10,720 |
| 25 | 16 | Hospital for Epilepsy, &c.... ... | 97 | 731 | 1 924 | 916 | 50 | 579 | 1,545 | 266 |
| 50 | 22 | West End, for Epilepsy, &c. ... | 133 | 2 210 | 2,410 | 1,531 | 363 | 445 | 2,339 | 2,888 |
| 13 | 6 | Central London Ophthalmic ... | [illegible] | 8 668 | 888 | 454 | 80 | 299 | 833 | ... |
| 10 | 2 | Western Ophthalmic ... ... | 54 | 2,478 | 608 | 472 | 124 | 165 | 761 | ... |
| 63 | 57 | National Orthopædic ... ... | 216 | 660 | 1,701 | 594 | ... | 1,092 | 1,686 | ... |
| 94 | 53 | London Homœopathic ... ... | 761 | 10,245 | 4,989 | 1,465 | 3,158 | 402 | 5,025 | 308 |
| 25 | 12 | Establishment for Gentlewomen ... | 112 | ... | 2,037 | 833 | 71 | 895 | 1,799 | ... |
| ... | ... | National Dental ... ... ... | ... | 23,847 | 701 | 786 | ... | 82 | 868 | ... |
| 1,363 | 1,036 | | 10,182 | 141,424 | 102,820 | 44,870 | 20,487 | 8,328 | 73,685 | 56,726 |
| | | DISPENSARIES. | | | | | | | | |
| ... | ... | Bloomsbury, Provident ... ... | ... | 537 | 306 | ... | ... | 268 | 268 | ... |
| ... | ... | London Medical Mission ... ... | ... | 3,117 | 1,374 | 1,528 | 42 | ... | 1,570 | ... |
| ... | ... | Margaret Street Infirmary... ... | ... | 2,420 | 536 | 300 | 182 | ... | 482 | ... |
| ... | ... | Portland Town ... ... ... | ... | 2,189 | 191 | 123 | 2 | 14 | 139 | ... |
| ... | ... | Portobello Road ... ... ... | ... | 220 | 81 | 64 | 4 | 57 | 125 | ... |
| ... | ... | St. John's Wood Provident ... | ... | 4 355 | 496 | 242 | 26 | 251 | 519 | ... |
| ... | ... | St. Marylebone General ... ... | ... | 3,869 | 836 | 364 | 136 | 223 | 723 | ... |
| ... | ... | Western General ... ... ... | ... | 21,131 | 1,445 | 1,087 | 35 | ... | 1,122 | 325 |
| 1,363 | 1,036 | | 10,182 | 179,262 | 108,085 | 48,578 | 20,914 | 9,141 | 78,633 | 57,051 |

### KENSINGTON AND WEST DISTRICT,

Comprising Kensington, Paddington, Bayswater, Kilburn, Chelsea, Brompton, Fulham, Hammersmith, Chiswick, Brentford, Acton, Ealing, &c.

| No. of Beds. | No. of Beds Daily Occupied. | HOSPITALS. | In-patients. | Out-patients. | Total Expenditure. | Income. Charitable. | Income. Proprietary. | Income. Patients' Payments. | Total Income. | Legacies not included in previous column. |
|---|---|---|---|---|---|---|---|---|---|---|
| | | | | | £ | £ | £ | £ | £ | £ |
| 335 | 225 | St. George's ... ... ... ... ... | 3,080 | 31,828 | 46,283 | 9,444 | 15,375 | ... | 24,819 | 6,440 |
| 281 | 246 | St. Mary's ... ... ... ... ... | 3,729 | 30,975 | 23,514 | 10,933 | 2,450 | ... | 13,383 | 10,557 |
| 101 | 92 | West London ... ... ... ... | 1,620 | 26,089 | 6,834 | 5,363 | 183 | ... | 5,546 | 3,615 |
| 321 | 270 | Hospital for Consumption... ... | 1,602 | 15,357 | 30,407 | 14,876 | 8,943 | ... | 23,819 | 12,973 |
| 24 | 21 | Belgrave, for Children ... ... | 196 | 2,611 | 1,901 | 1,281 | 24 | ... | 1,305 | ... |
| 50 | 50 | Cheyne, for Sick & Incurable Children | 72 | .. | 2,396 | 1,791 | 107 | 501 | 2,399 | 41 |
| 20 | 9 | Paddington Green, for Children ... | 137 | 10,837 | 2,370 | 1,894 | 157 | 160 | 2,211 | 389 |
| 116 | 95 | Victoria, for Children ... ... | 1,375 | 17,075 | 6,794 | 5,740 | 1,047 | 414 | 7,201 | 1,319 |
| 60 | 40 | Chelsea, for Women ... ... | 518 | 3,493 | 4,482 | 3,124 | 59 | 680 | 3,863 | 1,225 |
| 105 | 75 | Cancer ... ... ... ... | 811 | 1,289 | 10,401 | 5,821 | 1,984 | ... | 7,805 | 13,754 |
| 135 | 97 | Female Lock... ... ... ... | 706 | ... | 4 429 | 1,951 | 13 | 2,163 | 4,127 | 1,074 |
| 20 | 14 | Male Lock ... ... ... ... | 274 | 3,863 | 1,853 | 146 | ... | 1,833 | 1,979 | 200 |
| 30 | 28 | St. Mary Magdalene's Home ... | 60 | ... | 832 | 410 | 111 | 405 | 926 | ... |
| 1,598 | 1,262 | | 14,180 | 143,417 | 142,496 | 62 774 | 30,453 | 6,156 | 99,383 | 51,587 |
| | | DISPENSARIES. | | | | | | | | |
| ... | ... | Brompton Provident ... ... | ... | 1,077 | 497 | 153 | 79 | 266 | 498 | ... |
| ... | ... | Chelsea, &c. ... ... ... ... | ... | 5,104 | 710 | 509 | 176 | ... | 685 | ... |
| ... | ... | Kensal Town, Provident ... ... | ... | 689 | 305 | 49 | ... | 284 | 333 | ... |
| ... | ... | Kensington ... ... ... ... | ... | 4,122 | 663 | 614 | 47 | ... | 661 | 100 |
| ... | ... | Kilburn, Maida Vale ... ... | ... | 3,460 | 515 | 693 | 51 | ... | 744 | ... |
| ... | ... | Kilburn Provident ... ... ... | ... | 4,771 | 1,217 | 77 | 74 | 1,006 | 1,157 | ... |
| ... | ... | Notting Hill Provident ... ... | ... | 916 | 238 | 153 | ... | 87 | 240 | ... |
| ... | .. | Paddington Provident ... ... | ... | 7,394 | 566 | 183 | 24 | 369 | 576 | 100 |
| ... | ... | Pimlico Provident ... ... ... | ... | 2,732 | 518 | 21 | ... | 516 | 527 | ... |
| ... | ... | Royal Pimlico Provident ... ... | ... | 11,609 | 894 | 344 | 16 | 555 | 915 | ... |
| ... | ... | Westbourne Provident ... ... | ... | 1,030 | 380 | 59 | 43 | 300 | 402 | ... |
| 1,598 | 1,262 | | 14 180 | 186 321 | 148,999 | 65 629 | 30,963 | 9,539 | 106,131 | 51,787 |

### ISLINGTON AND NORTH-WEST DISTRICT,

Comprising Islington, Holloway, Highbury, Hampstead, Highgate, St. Pancras, Stoke Newington, Tottenham, &c.

| No. of Beds. | No. of Beds Daily Occupied. | HOSPITALS. | In-patients. | Out-patients. | Total Expenditure. | Income. Charitable. | Income. Proprietary. | Income. Patients' Payments. | Total Income. | Legacies not included in previous column. |
|---|---|---|---|---|---|---|---|---|---|---|
| | | | | | £ | £ | £ | £ | £ | £ |
| 72 | 68 | Great Northern Central ... ... | 1,030 | 22,570 | 5,985 | 3,774 | 711 | ... | 4 485 | 16,902 |
| 44 | 39 | North-West London ... ... | 555 | 14,159 | 4 012 | 4,401 | 162 | 73 | 4,636 | 420 |
| 103 | 63 | Tottenham ... ... ... ... | 845 | 8,687 | 4,963 | 2,998 | 932 | 971 | 4,901 | ... |
| 207 | 183 | University College ... ... ... | 2,799 | 36 999 | 20,108 | 7,177 | 4,998 | 18 | 12,193 | 24,737 |
| 50 | 45 | North London Consumption ... | 371 | 2,899 | 5,576 | 4,936 | 149 | 17 | 5,102 | 3,939 |
| 170 | 98 | London Fever ... ... ... | 748 | ... | 9,634 | 7,483 | 2 318 | 2,564 | 12,365 | 4,157 |
| 120 | 70 | London Temperance ... ... | 859 | 8,190 | 8,033 | 4,589 | 1,049 | 170 | 5 808 | 2,326 |
| 28 | 12 | Hampstead Home Hospital ... | 184 | ... | 2,538 | 1,290 | 813 | 525 | 2,628 | 100 |
| 28 | 18 | Invalid Asylum ... ... ... | 217 | ... | 877 | 558 | 153 | 200 | 911 | ... |
| 822 | 596 | | 7,008 | 93,504 | 61,726 | 37,206 | 11,285 | 4,538 | 53,029 | 52,381 |
| | | DISPENSARIES. | | | | | | | | |
| ... | ... | Camden Provident ... ... ... | ... | 964 | 301 | 13 | ... | 243 | 256 | ... |
| ... | ... | Child's Hill ... ... ... ... | ... | 4,202 | 266 | 40 | 11 | 220 | 271 | 200 |
| ... | ... | Hampstead Provident ... ... | ... | 10,640 | 944 | 309 | 20 | 624 | 953 | ... |
| ... | ... | Holloway and North Islington ... | ... | 4,101 | 888 | 378 | 23 | 354 | 755 | 200 |
| ... | ... | Islington ... ... ... ... | ... | 9,896 | 827 | 296 | 29 | 497 | 822 | 22 |
| ... | ... | St. Pancras and Northern ... ... | ... | 4,899 | 645 | 280 | 103 | 85 | 468 | 45 |
| ... | ... | Stamford Hill, &c. ... ... ... | ... | 5,211 | 657 | 431 | 137 | ... | 568 | ... |
| 822 | 596 | | 7,008 | 133,417 | 66,254 | 38,953 | 11,608 | 6,561 | 57,122 | 52,848 |

### WESTMINSTER DISTRICT,

Comprising Westminster City and Liberties.

| No. of Beds. | No. of Beds Daily Occupied. | HOSPITALS. | In-patients. | Out-patients. | Total Expenditure. | Income. Charitable. | Income. Proprietary. | Income. Patients' Payments. | Total Income. | Legacies not included in previous column. |
|---|---|---|---|---|---|---|---|---|---|---|
| | | | | | £ | £ | £ | £ | £ | £ |
| 175 | 110 | Charing Cross ... ... ... | 2,007 | 16,312 | 16,432 | 5,250 | 3,165 | ... | 8 415 | 8.224 |
| 220 | 184 | King's College ... ... ... | 2,212 | 24,265 | 19,510 | 8 837 | 2 424 | 85 | 11,346 | 6 329 |
| 205 | 186 | Westminster ... ... ... ... | 2,989 | 26,002 | 13,480 | 4,104 | 3,301 | ... | 7,405 | 12,403 |
| 134 | 109 | Ventnor, for Consumption ... | 747 | ... | 11,837 | 3,979 | 1,799 | 3,258 | 9,036 | 4,469 |
| 18 | 12 | Grosvenor, for Women & Children | 130 | 1,436 | 1,505 | 961 | 16 | 344 | 1,321 | 25 |
| 64 | 44 | Hospital for Women ... ... | 580 | 5,153 | 5,742 | 2,670 | 269 | 828 | 3,767 | 17,592 |
| 25 | 22 | National, for Diseases of Heart, &c. | 108 | 1,762 | 2,085 | 1,677 | 46 | 343 | 2,066 | ... |
| 30 | 26 | Royal Westminster Ophthalmic ... | 388 | 8,591 | 2,272 | 1,558 | 379 | 51 | 1,988 | 109 |
| 50 | 47 | Royal Orthopædic ... ... ... | 171 | 1,071 | 2,238 | 1,291 | 524 | 175 | 1,990 | 269 |
| 25 | 21 | Hospital for Diseases of the Throat | 633 | 7,941 | 3,590 | 535 | 201 | 2,662 | 3 398 | 100 |
| 9 | 6 | Royal Ear ... ... ... ... | 179 | 2,596 | 887 | 296 | 11 | 595 | 902 | ... |
| ... | ... | Dental ... ... ... ... | ... | 50,480 | 2,190 | 2,096 | 198 | 443 | 2,737 | ... |
| 11 | 9 | Gordon, for Fistula ... ... ... | 134 | 526 | 1,069 | 599 | 30 | 539 | 1,168 | ... |
| 26 | 15 | St. Peter's, for Stone ... ... | 367 | 7,350 | 3,461 | 681 | 492 | 1,852 | 3,025 | 50 |
| 992 | 791 | | 10,645 | 153,485 | 86,298 | 34,534 | 12,855 | 11,175 | 58,564 | 49,570 |
| | | DISPENSARIES. | | | | | | | | |
| ... | ... | Public ... ... ... ... ... | ... | 2,977 | 645 | 445 | 160 | ... | 605 | ... |
| ... | ... | St. George and St. James ... ... | ... | 3,436 | 560 | 441 | 14 | 35 | 490 | 200 |
| ... | ... | St. George, Hanover Square ... | ... | 1,849 | 647 | 416 | 22 | 141 | 579 | ... |
| ... | ... | Western ... ... ... ... | ... | 3,592 | 1,302 | 403 | 394 | 461 | 1,258 | ... |
| ... | ... | Westminster General ... ... | ... | 4,428 | 436 | 286 | 143 | 57 | 486 | ... |
| 992 | 791 | | 10,645 | 169.767 | 89,888 | 36,525 | 13,588 | 11,869 | 61,982 | 49,770 |

### STRATFORD AND EAST-END DISTRICT,

Comprising Bethnal Green, Tower Hamlets, West Ham, Whitechapel, Hackney, Stepney, Limehouse, Poplar, and the East.

| No. of Beds. | No. of Beds Daily Occupied. | HOSPITALS. | In-patients. | Out-patients. | Total Expenditure. | Income. Charitable. | Income. Proprietary. | Income. Patients' Payments. | Total Income. | Legacies not included in previous column. |
|---|---|---|---|---|---|---|---|---|---|---|
| | | | | | £ | £ | £ | £ | £ | £ |
| 125 | 94 | German ... ... ... ... | 1,283 | 24,113 | 10,147 | 6,089 | 2,383 | 343 | 8,815 | 2,043 |
| 776 | 630 | London ... ... ... ... | 9,409 | 112,962 | 73,939 | 22,597 | 23,716 | 569 | 46,882 | 18,391 |
| 42 | 31 | Poplar ... ... ... ... | 633 | 13,200 | 7,618 | 3.048 | 788 | ... | 3,836 | 3,545 |
| 164 | 112 | City of London for Dis. of the Chest | 1,079 | 14,865 | 11,150 | 6 684 | 388 | ... | 7,072 | 5,817 |
| 102 | 77 | East London for Children ... ... | 1,257 | 7,296 | 8,730 | 6,273 | 498 | ... | 6,771 | 4,090 |
| 93 | 53 | Mrs. Gladstone's Home ... ... | 983 | ... | 1,270 | 596 | 363 | ... | 959 | 500 |
| 12 | 6 | East-End Mothers' Home ... ... | 140 | 201 | 1,058 | 1,174 | 94 | 38 | 1,306 | ... |
| 37 | 23 | West Ham, &c. ... ... ... | 348 | 24,555 | 2,915 | 2,792 | ... | ... | 2,792 | 242 |
| ,351 | 1,026 | | 15,132 | 197,192 | 116,827 | 49,253 | 28,230 | 950 | 78,433 | 34,628 |
| | | DISPENSARIES. | | | | | | | | |
| ... | ... | Eastern ... ... ... ... | ... | 7,189 | 762 | 344 | 301 | 66 | 711 | ... |
| ... | ... | Hackney Provident ... ... ... | ... | 631 | 267 | 28 | ... | 243 | 271 | ... |
| ... | ... | London ... ... ... ... | ... | 2,320 | 426 | 138 | 266 | ... | 404 | 100 |
| ... | ... | Queen Adelaide ... ... ... | ... | 4,314 | 509 | 434 | 196 | 48 | 678 | ... |
| ... | ... | Tower Hamlets ... ... ... | ... | 4,705 | 691 | 689 | 26 | 128 | 843 | 50 |
| 1,351 | 1,026 | | 15.132 | 216,351 | 119,572 | 50,886 | 29,019 | 1,435 | 81,340 | 34,778 |

### SUMMARY.

It will be seen from the following summary that the Voluntary Hospitals and Medical Charities of London, during the 12 months ending 31st December, 1892, relieved over one million two hundred and seventy-two thousand patients at a cost of £677,724. The Ordinary Income only amounted to £505,843, leaving a deficiency of £171,881 on the year's work.

| No. of Beds. | No. of Beds Daily Occupied. | Hospitals and Dispensaries. | In-patients. | Out-patients. | Total Expenditure. | Income. Charitable. | Income. Proprietary. | Income. Patients' Payments. | Total Income. | Legacies not included in previous column. |
|---|---|---|---|---|---|---|---|---|---|---|
| | | | | | £ | £ | £ | £ | £ | £ |
| 1,468 | 1,018 | Newington and South District ... | 14,126 | 166,228 | 90,896 | 36,649 | 37,030 | 10,820 | 84 499 | 3,555 |
| 586 | 436 | City and East Central District ... | 6,899 | 142 773 | 54,030 | 21,225 | 10,526 | 4,385 | 36,136 | 27,082 |
| 992 | 791 | Westminster District ... ... | 10,645 | 169,767 | 89,888 | 36,525 | 13,588 | 11,869 | 61,982 | 49,770 |
| 1,363 | 1,036 | St. Marylebone and West Central District... ... ... ... | 10,182 | 179,262 | 108,085 | 48,578 | 20,914 | 9,141 | 78,633 | 57,051 |
| 1,598 | 1,262 | Kensington and West District ... | 14,180 | 186 321 | 148 999 | 65,629 | 30,963 | 9,539 | 106,131 | 51,787 |
| 822 | 596 | Islington and North-West District | 7,008 | 133,417 | 66,254 | 38,953 | 11,608 | 6,561 | 57,122 | 52,848 |
| 1,351 | 1,026 | Stratford and East-End District... | 15,132 | 216,351 | 119,572 | 50,886 | 29,019 | 1,435 | 81,340 | 34,778 |
| 8,180 | 6,165 | | 78,172 | 1,194,119 | 677,724 | 298,445 | 153,648 | 53,750 | 505,843 | 276,871 |

### HOSPITAL FOR SICK CHILDREN, GREAT ORMOND STREET.

### SCRAPS AND GLEANINGS.

Paris is shortly to be provided with a new hospital for phthisis.

The Woman's Medical College at Chicago has graduated a class of 30.

The average number of births in London workhouses is estimated at about 1.000.

The Edinburgh School of Medicine for Women is presided over by the Duchess of Fife.

The opening of the cottage hospital at Malmesbury took place the second week in May.

An infectious diseases hospital is considered necessary for the district of Sedgley, near Dudley.

The Wallsall Cottage Hospital at a meeting stated that 1,697 patients had been admitted during the last quarter.

The Dee Oil Company (Limited) have removed to 49, Lime Street, E.C., during rebuilding of the premises at 38, Leadenhall Street.

The Manchester Unity of Oddfellows at Southampton the other day, decided by a large majority to admit of females joining the order.

Professor Wilhelm Erl, of Heidelberg, has been called to Vienna to occupy the vacant seat of clinical medicine, vacated by Dr. Kahler.

The Corporation of the City of London have made a grant of £105 towards the funds of the Metropolitan Hospital, Kingsland Road, N.E.

Newcastle is going shortly to erect municipal lodging houses, which will be built on land belonging to the corporation, at a cost of about £27,000.

A series of dramatic performances were recently given in the City Hall, Perth, to collect funds towards the Perth Sick Poor Nursing Society.

The initial steps taken by the Sanitary Authority for Luton Union to provide an infectious diseases hospital have raised a storm of indignation.

The first steps have been taken towards the demolition of a well-known London hospital, the London Homœopathic, in Great Ormond Street, Queen Square.

The National College for the Deaf at Pennsylvania has graduated two women students. The last of the two successful competitors carried off the honours of her class.

A children's convalescent home has been recently acquired about two miles south of Newport, Dundee. It is a two-storeyed house, with numerous outbuildings.

The Mineral Water Hospital at Bath presents a satisfactory report for the past year. The Hospital continues steadily to increase in importance and usefulness.

The representatives of the Cockermouth Local Board and Rural Sanitary Authority are in treaty for the purchase of a farm house, to be used as an infectious diseases hospital.

The money received in Church collections for the cottage hospital at Uckfield was £4, and the street collection at the Hospital Sunday collection for the same reached the sum of £5.

At the 20th annual meeting of the North Cambridgeshire Hospital, the report stated that the income account showed a balance of £351. There had been 130 cases admitted during the year.

The Whitworth Hospital at Drumcondra showed a slight balance to the good at the annual meeting. Subscriptions and donations to the extent of £560 have been received during the year.

The congress of German philologists has held its forty-second meeting at Vienna. A special English section was appointed this year. The list of members is greatly on the increase to this important body.

The Daisy Home for Children, Bexhill on-Sea, shows, in its annual report, an improvement on the balance-sheet of accounts. The total receipts for the year amount to £152, and the expenditure to £148.

A large sale of work was held recently at Bury, all of which articles were made by the inmates of the Robinson Kay Home for Females, which is a branch of the parent institution, the Home for Incurables.

The twenty-third annual meeting of the subscribers to the Wimbledon Cottage Hospital was held in May, and the statement of accounts shows that the financial year was begun with a balance in hand of £83.

The Malvern Rural Hospital reports that during the year 1892 96 cases have been treated. The expenses of the hospital had not quite been covered by the income, though the deficiency is only a few pounds.

It is announced that the Duchess of Teck and Princess May have promised to attend the annual sale of the inmates' work at the Royal Hospital for Incurables, to be held within that establishment on June 6th.

The Hospital Saturday collection at Birmingham for this year shows a decided increase in funds. The total collection for the year from 987 firms reached the sum of £900, whilst the street collections contributed another £330.

The will of the late Katherine Page Perkins endows Harvard College with a legacy of 150,000 dollars. This money will be applied for the erection of a dormitory to be known as Perkins Hall, as a memorial to the late Miss Perkins' family.

Westminster Hospital Athletic Club will hold their fourth annual meeting at Stamford Bridge Grounds, Walham Green, on Friday, June 9th, commencing 2.30 p.m. The entries this year are more numerous, and a good day's sport is anticipated.

Mr. Charles Tweedy has lately been presented with a gold watch and chain and a silver salver, in recognition of his services as hon. secretary to the Miner's Hospital, Redruth, a great number of subscribers and friends aiding in this presentation.

The Committee of the North Shields and Tynemouth Dispensary show a balance on the year's account of £145. The Committee further stated, at the annual meeting, that it is suggested to sell the present building and erect a new one in a more central position.

A favourable report on the year's proceedings was presented at the annual meeting of the Devizes Cottage Hospital. The financial statement of accounts shows receipts to the amount of £668, and the payments £664, the annual subscription from the town being £107.

### Medical Vacancies and Appointments.

### VACANCIES.

Following vacancies are announced this week, the amount of in each case being given after the name of the institution:

#### Resident Medical Officers.

Central London Ophthalmic Hospital, 238A, Gray's Inn Road, W.C. House-Surgeon. Applications to the Secretary by June 6th.

City of London Hospital for Diseases of the Chest, Victoria Park, E. House-Physician. Appointment for six months. Board, residence, and allowance for washing provided. Applications to the Secretary, at the office, 24, Finsbury Circus, E.C., by June 8th.

East London Hospital for Children, Glamis Road, Shadwell, E. House-Surgeon. Board and lodging provided. Applications to the Secretary by July 6th.

General Infirmary, Northampton. House-Surgeon; doubly qualified, unmarried, and not under 23 years of age. Salary £125 per annum, with furnished apartments, board, attendance, and washing. The Assistant House-Surgeon is a candidate, and in the event of his appointment the Court will proceed to the election of an Assistant House-Surgeon; Salary £80 per annum, furnished apartments board, attendance, and washing. Applications to the Secretary by June 10th.

Hospital for Sick Children, Newcastle-on-Tyne. Resident Medical Officer; doubly qualified. Salary £60 per annum, with board, lodging, and laundry. Applications to Robert J. Gibson, Secretary, by June 5th.

North-Eastern Hospital for Children, Hackney Road, N.E. Junior House-Surgeon, doubly qualified. Appointment for six months. Salary at the rate of £60 per annum. Applications to Alfred Nixon, Secretary, 27, Clement's Lane, E.C., by June 10th.

Royal Free Hospital, Gray's Inn Road, W.C. Junior Resident Medical Officer. House-Surgeon; doubly qualified. Appointment for six months. Board, residence, and washing provided. Applications to the Secretary by June 19th.

Wolverhampton and Staffordshire General Hospital, Wolverhampton. Resident Assistant. Appointment for six months. Board, lodging, and washing provided. Applications, inscribed "Applications for Resident Assistant," to the Chairman of the Medical Committee by June 5th.

#### Non-Resident Medical Officers.

Chelsea Hospital for Women, Fulham Road, S.W. Physician to out-patients. Applications (on forms to be obtained) to A. C. Davis, Secretary, by June 5th.

Hospital for Consumption and Diseases of the Chest, Brompton. Assistant Physician. Applications to Henry Dobbin, Secretary, by June 7th.

Martley Union. Medical Officer for the Leigh District. Salary £55 per annum, and extra fees prescribed by the Local Government Board. Applications to A. W. Knott, Clerk, at the Board Room, Martley, by June 8th.

Metropolitan Hospital, Kingsland Road, N.E. Ophthalmic Surgeon. Must be F.R.C.S.Eng. Applications to Charles H. Byers, Secretary, by June 9th.

Royal Infirmary, Bristol. Honorary Assistant Physician. Applications and testimonials to J. F. Shekleton, Secretary and House Governor (who can forward a list of the Committee of Election), by June 10th.

Sunderland Infirmary. Honorary Medical Officer to the Convalescent House at Heatherdene, Harrogate; doubly qualified. Applications to the Chairman of the Medical Board by June 6th.

Victoria Hospital for Sick Children, Queen's Road, Chelsea, S.W. Surgeon on the in-patient staff and Surgeon on the out-patient staff. Appointment for five years. Applications to Commander Blount, R.N., Secretary, by June 17th.

### PASS LIST.

#### ROYAL COLLEGE OF SURGEONS IN IRELAND.

Election of Examiners.—The following gentlemen have been elected examiners for for the ensuing year: Anatomy and Surgery: John Barton, William Stoker, Sir William Stokes, William Thomson. Physiology and Histology: J. Alfred Scott. Biology: J. Alfred Scott. Chemistry and Physics: Robert J. Montgomery. Midwifery and Gynæcology: Samuel R. Mason. Ophthalmology: Arthur Henry Benson, Patrick Wm. Maxwell. Dentistry: John Barton, John J. Burgess, William Stoker, Henry Gregg Sherlock, Thomas Studley, Charles Wall. Diploma in State Medicine: Hugh A. Auchinleck, D. Edgar Flinn, William Stoker (Morbid Anatomy), P. Crampton Smyly. Midwifery Diploma: Richard Thomas Hearn, Samuel R. Mason, Jeremiah O'Donovan. General Education: Frank J. Davys, Robert Morton.

